# Magnolol, a Natural Polyphenol, Attenuates Dextran Sulfate Sodium-Induced Colitis in Mice

**DOI:** 10.3390/molecules22071218

**Published:** 2017-07-20

**Authors:** Ling Zhao, Hai-tao Xiao, Huai-xue Mu, Tao Huang, Ze-si Lin, Linda L. D. Zhong, Guang-zhi Zeng, Bao-min Fan, Cheng-yuan Lin, Zhao-xiang Bian

**Affiliations:** 1 Lab of Brain and Gut Research, School of Chinese Medicine, Hong Kong Baptist University, Kowloon Tong, Hong Kong, China; zhangda0525@163.com (L.Z.); xiaoht81@163.com (H.-t.X.); mudandan49@163.com (H.-x.M.); thuangsh@gmail.com (T.H.); lin.zesi@163.com (Z.-s.L.); ldzhong0305@gmail.com (L.L.D.Z.); 2 Hong Kong Baptist University Shenzhen Research Institute and Continuing Education, 518057 Shenzhen, China; 3 Preparatory Office of Shenzhen-Melbourne Institute of Life Sciences and Bioengineering, Guangzhou University of Chinese Medicine, 510006 Guangzhou, China; 4 YMU-HKBU Joint Laboratory of Traditional Natural Medicine, Yunnan Minzu University, 650500 Kunming, China; g.zh_zeng@163.com (G.-z.Z.); adams.bmf@hotmail.com (B.-m.F.)

**Keywords:** magnolol, inflammation, ulcerative colitis, tryptophan metabolites

## Abstract

Magnolol is a lignan with anti-inflammatory activity identified in *Magnolia officinalis*. Ulcerative colitis (UC), one of the types of inflammatory bowel disease (IBD), is a disease that causes inflammation and ulcers in the colon. To investigate the effect of magnolol in dextran sulfate sodium (DSS)-induced experimental UC model, male C57 mice were treated with 2% DSS drinking water for 5 consecutive days followed by intragastric administration with magnolol (5, 10 and 15 mg/kg) daily for 7 days. The results showed that magnolol significantly attenuated disease activity index, inhibited colonic shortening, reduced colonic lesions and suppressed myeloperoxidase (MPO) activity. Moreover, colonic pro-inflammatory cytokines (TNF-α, IL-6, and IL-1β) induced by colitis were dramatically decreased by magnolol. To further unveil the metabolic signatures upon magnolol treatment, mass spectrometry-based metabolomic analysis of the small molecular metabolites in mice serum were performed. Compared with controls, abnormality of serum metabolic phenotypes in DSS-treated mice were effectively reversed by different doses of magnolol. In particular, magnolol treatment effectively elevated the serum levels of tryptophan metabolites including kynurenic acid (KA), 5-hydroxyindoleacetic acid, indoleacetic acid (IAA), indolelactic acid and indoxylsulfuric acid, which are potential aryl hydrocarbon receptor (AHR) ligands to impact colitis. These findings suggest that magnolol exerts anti-inflammatory effect on DSS-induced colitis and its underlying mechanisms are associated with the restoring of tryptophan metabolites that inhibit the colonic inflammation.

## 1. Introduction

Inflammatory bowel disease (IBD), sub-grouped by ulcerative colitis (UC) and Crohn’s disease (CD), is a chronic relapsing inflammatory disorder characterized by diffuse mucosal inflammation, granuloma/crypt abscess, and infiltration of neutrophils in the colon [1]. The incidence of IBD is traditionally high in North America and Europe, and rising rapidly in Asia, paralleling westernization [2]. Unfortunately, the current treatments for IBD are not completely satisfactory. 5-aminosalicylates (5-ASA) and corticosteroids are commonly used in mild-to-moderate UC patients, while long-term treatment with these drugs are not quite effective [3,4]. Other steroid drugs such as azathioprine and 6-mercaptopurine exist non-negligible side effects [5,6]. Infliximab is the most often used biological agent for IBD, while the clinical response is only around 65% [7]. To date, the underlying cause of IBD is not well-understood and alarmingly, in light of the soaring numbers of IBD cases, promising therapeutics have yet to be developed.

Magnolol (5,5′-di-2-propen-1-yl-2,2′-Bichavicol) is a lignan exacted from the bark of *Magnolia officinalis*, the compounds of which demonstrating anti-cancer, anti-stress, anti-anxiety, anti-depressant, anti-oxidant and anti-inflammatory effects [8]. The anti-inflammatory effect of magnolol has been reported in the animal models of ischemic stroke [9], oxygen-induced retinopathy [10], acute lung injury [11,12], mastitis [13], and arthritis [14]. In RAW264.7 cells, magnolol can inhibit lipopolysaccharide (LPS)-induced NF-κB activation, IKBα degradation and pro-inflammatory cytokines production [15,16]. Similar mechanisms of magnolol can also be found in LPS-induced acute lung injury mice models by inhibiting the tumor necrosis factor-alpha (TNF-α), IL-1β, phosphorylation of IκB-α and NF-κB, and expression of toll-like receptor 4 (TLR4) [17]. In human aortic endothelial cells, magnolol inhibits TNF-α-induced JNK/p38 phosphorylation and NF-κB activation, thereby reducing leukocyte adhesion [18]. Furthermore, magnolol was demonstrated to suppress colonic smooth muscle contraction through downregulation of l-type Ca(2+) channels in rats [19,20]. Magnolol treatment prevents sepsis-induced rat intestinal dysmotility by regulating stem cell factor (SCF)/c-kit and NO signaling to maintain functional interstitial cells of Cajal (ICCs) [21], reducing TNF-α and inducible nitric oxide synthase (iNOS), while stimulating IL-10 mRNA expression in septic rat ileum [22]. These findings suggest that magnolol is a potential therapeutic compound for inflammatory gastrointestinal diseases yet to be examined. The present study aims to investigate the effect of magnolol on Dextran sulfate sodium (DSS)-induced colitis, and to explore the possible mechanisms in the levels of metabolites by UPLC/MS-based metabolomic analysis.

## 2. Results

### 2.1. Magnolol Impact the Severity of DSS-Induced Colitis in Mice

**Figure 1:**
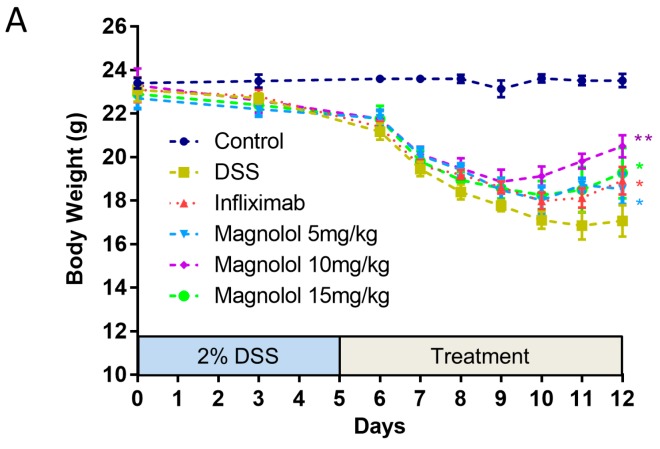

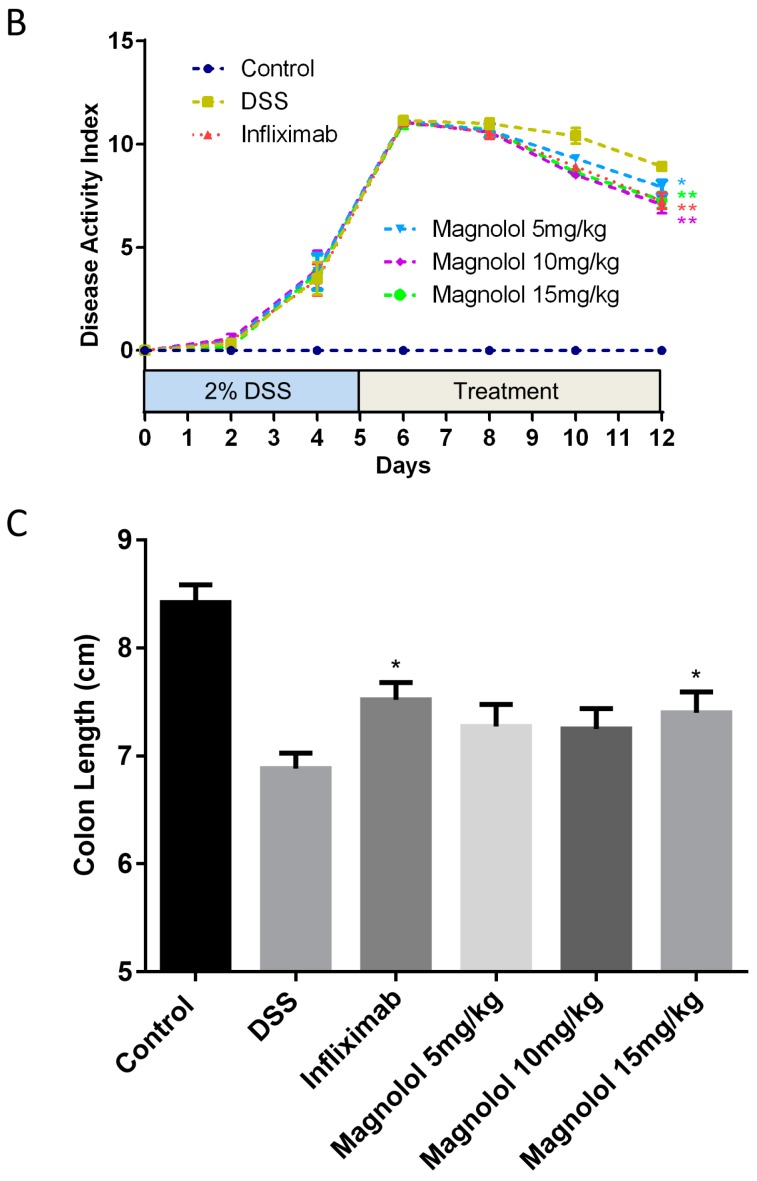
The phenotypic severity of dextran sulfate sodium (DSS)-induced colitis in mice can be effectively attenuated by one-week treatment of magnolol. (**A**) The body weight loss was significantly improved in all treatment groups versus DSS group; (**B**) The enhanced disease activity index of DSS mice was significantly reduced in all treatment groups; (**C**) DSS-induced shortened colon was significantly improved by high dose of magnolol and infliximab. The value in the plot was expressed as means ± SEM, and statistically significant was marked by asterisk (* *p* < 0.05; ** *p* < 0.01, vs. DSS group).

Male C57BL/6J mice were induced with treatment of drinking water containing 2% DSS to develop typical symptoms of IBD including body weight loss, diarrhea and rectal bleeding. The effects of magnolol were evaluated after daily administration on the mice with colitis. As shown in Figure 1A, body weight dramatically dropped by DSS induction compared to vehicle group. The body loss caused by DSS treatment was significantly improved in mice receiving infliximab and magnolol. Medium dose of magnolol (10 mg/kg) get the best effect on body weight index. DSS administration for 5 days resulted in severe diarrhea, blood in stool and body weight loss integrated as remarkably high disease activity index (DAI). Daily administration of magnolol at doses of 5–15 mg/kg significantly reduced these effects compared with DSS group (Figure 1B). In addition, the mean lengths of the colon in mice treated with DSS were statistically shorter than that of control group. Magnolol (15 mg/kg) and infliximab treatment significantly improved the symptoms (Figure 1C).

### 2.2. Effects of Magnolol on Histopathological Changes and Myeloperoxidase Activity in the Colon of DSS-Treated Mice

**Figure 2:**
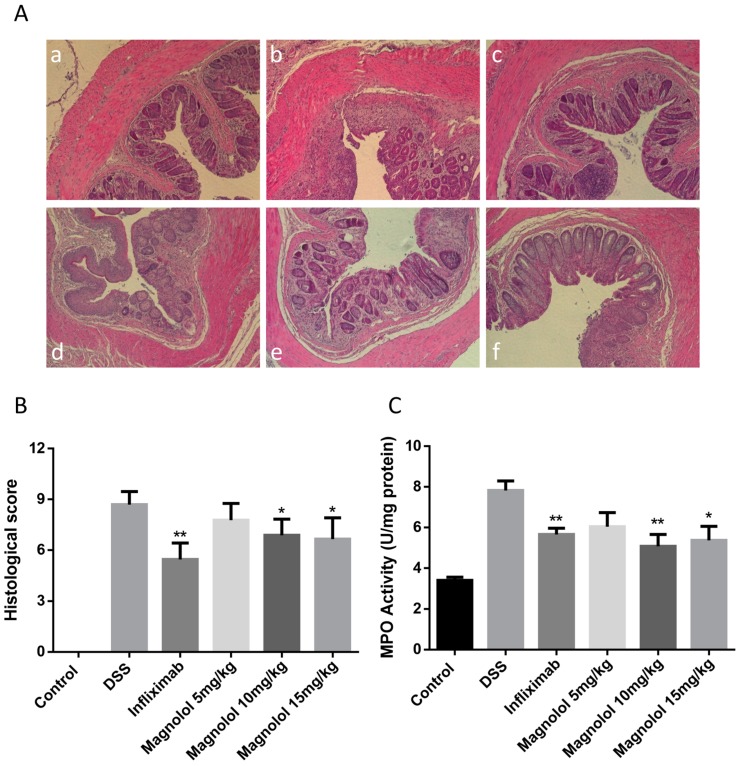
Medium and high dosages of magnolol effectively attenuated histopathological changes and myeloperoxidase activity in the colon of DSS-treated mice. (**A**) Representative images of hematoxylin/eosin (H&E) staining (magnification, 10×): (**a**) Control group; (**b**) DSS group; (**c**) Infliximab group; (**d**) Magnolol 5 mg/kg; (**e**) Magnolol 10 mg/kg; (**f**) Magnolol 15 mg/kg; (**B**) Histological scores; (**C**) MPO activity. The value in the plot was expressed as means ± SEM, and statistically significant was marked by asterisk (* *p* < 0.05; ** *p* < 0.01).

In mice, the diarrhea and stool blood caused by daily DSS treatment are accompanied by colonic inflammation and damage to the intestinal wall. As shown in Figure 2A, severe crypt destruction and inflammatory cell infiltration were observed in the histological sections from DSS-treated mice. Both magnolol and infliximab could attenuate the histopathological manifestation of colitis. The histological scores were also substantially decreased in the magnolol-treated groups and positive control group (Figure 2B). To evaluate the inflammatory cell infiltration in the colon, myeloperoxidase (MPO) activity in mice colonic tissues was examined. Consistent with histological scores, MPO activity in the proximal colon of DSS model group was 2-fold higher than the control group, which was significantly suppressed in the magnolol-treated and infliximab-treated group (Figure 2C).

### 2.3. Effects of Magnolol on Colonic Pro-Inflammatory Cytokines in DSS-Treated Mice

**Figure 3:**
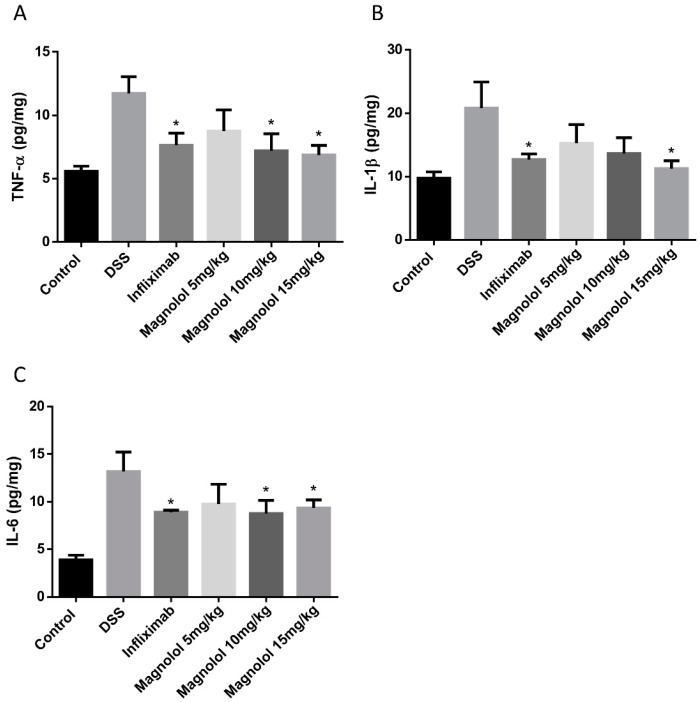
Medium and/or high dosages of magnolol significantly attenuated DSS-induced high levels of proinflammatory cytokines TNF-α (**A**), IL-1β (**B**) and IL-6 (**C**) in the colonic tissues. The value in the plot was expressed as means ± SEM, and statistically significant was marked by asterisk (* *p* < 0.05).

To test the anti-inflammatory effect of magnolol on DSS-induced colitis in mice, enzyme-linked immunosorbent assay (ELISA) was performed in mice colonic samples of all the experimental groups. The results reconstructed the cases that DSS significantly evoked the production of pro-inflammatory cytokines including TNF-α (Figure 3A), IL-1β (Figure 3B) and IL-6 (Figure 3C) in mice colons. As desired, these stimulatory effects could be attenuated by both infliximab and magnolol treatments at the dose of 10–15 mg/kg (Figure 3).

### 2.4. Effect of Magnolol on Alterations of Serum Metabolome in DSS-Treated Mice

**Figure 4:**
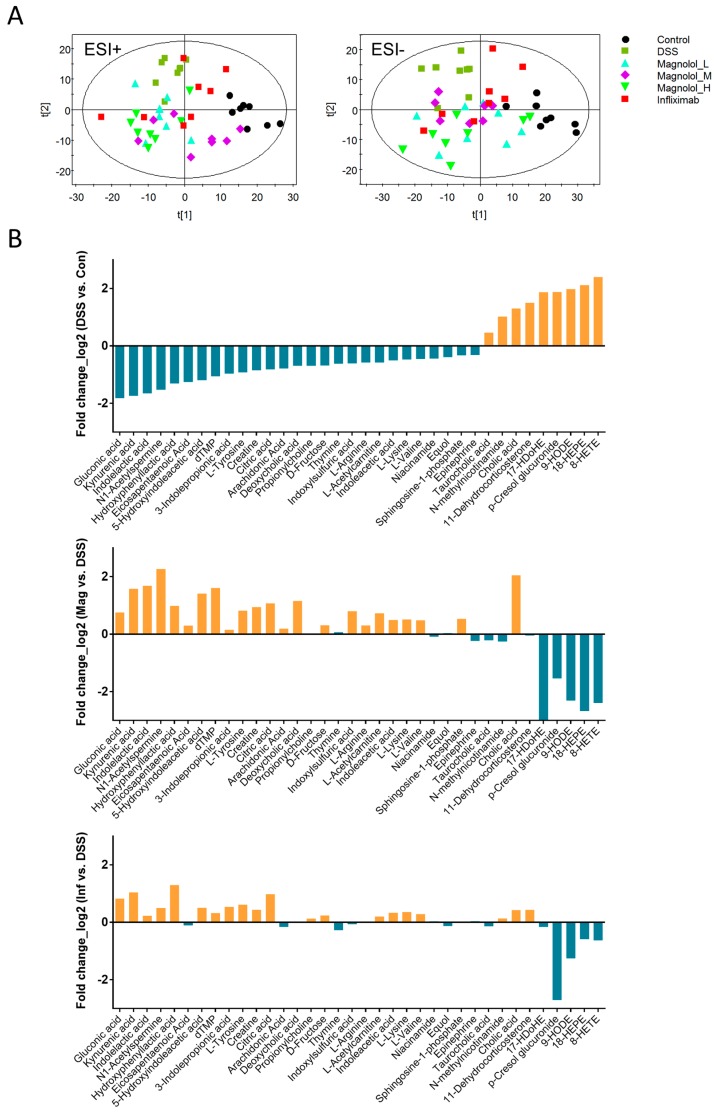
Magnolol majorly reversed abnormality of serum metabolome in colitis mice. (**A**) Two dimensional partial least squares discriminant analysis (PLS-DA) scatter plots displayed distinct metabolic profiles among model mice with and without drug treatment through UPLC/MS-based serum metabolomic analysis in both ESI modes. The variables explained 23% (t1) and 13.6% (t2) in ESI positive mode, while the variables explained 20.9% (t1) and 10.9% (t2) in ESI negative mode. (**B**) The log2 fold changes of all identified metabolic features between groups of DSS and control, groups of medium dose of magnolol and DSS as well as groups of infliximab and DSS.

As shown in the scatter plots of partial least squares discriminant analysis (PLS-DA), the serum metabolic profiles of DSS mice are notably distinct from that of controls using the first two principle components in both positive and negative ESI modes (Figure 4A). Meanwhile, serum metabolomes of treatment groups obviously deviated away from model group, indicating magnolol and infliximab both altered metabolic phenotype of DSS mice (Figure 4A). Particularly, in positive mode of ESI, the metabolic cluster of those mice treated with 10 mg/kg magnolol was closer to that of controls, demonstrating a smaller difference in serum metabolome between groups of magnolol (10 mg/kg) and control. In total, 34 metabolic features majorly contributing to serum phenotype of DSS mice were identified (Table 1 and Figure 4B), and majority of these metabolites were reversed by magnolol or/and infliximab (Table 2 and Figure 4B). There are 5 metabolites (citric acid, 9-HODE, hydroxyphenyllactic acid, gluconic acid and kynurenic acid) significantly reversed by both magnolol and infliximab, and 2 metabolites (cholic acid and p-cresol glucuronide) were regulated by infliximab only. However, compared with infliximab, magnolol significantly modulated more metabolites, in which lysine was regulated in all magnolol groups while another 17 metabolites were notably reversed in at least one dose of magnolol group. Particularly, medium dosage of magnolol displayed a broader effect of metabolic modulation than the other two dosages.

### 2.5. Magnolol Regulated Tryptophan Metabolic Pathway in Mice

**Figure 5:**
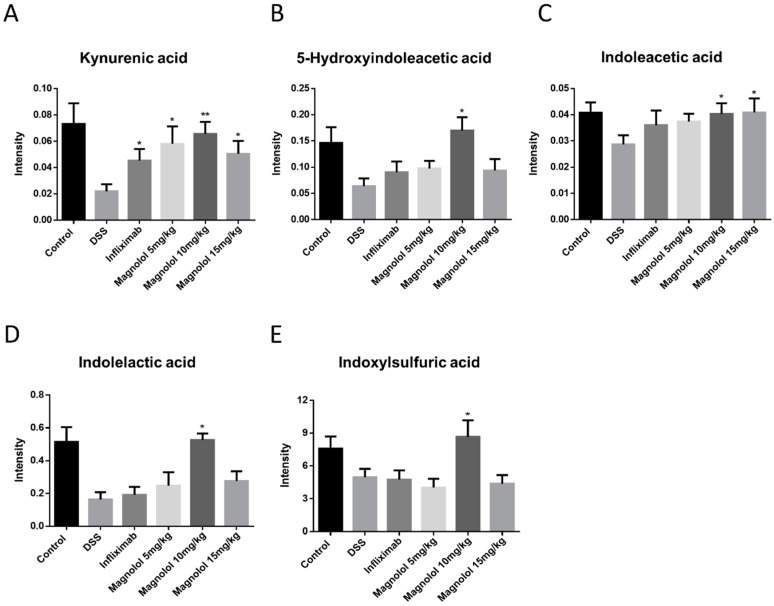
Reduction of serum metabolites kynurenic acid (**A**), 5-hydroxyindoleacetic acid (**B**), indoleacetic acid (**C**), indolelactic acid (**D**) and indoxylsulfuric acid (**E**) involved in tryptophan metabolism was significantly raised by magnolol treatment. The value in the plot was expressed as means ± SEM, and statistically significant was marked by asterisk (* *p* < 0.05; ** *p* < 0.01).

Referring to the databases of Kyoto Encyclopedia of Genes and Genomes (KEGG) and The Small Molecule Pathway Database (SMPDB), these DSS-associated metabolic changes were categorized into 19 metabolic pathways, majorly including tryptophan metabolism, arginine and proline metabolism, bile acid biosynthesis, unsaturated fatty acid synthesis, arachidonic acid metabolism, and microbial biotransformation (Table 1). Remarkably, DSS-induced reduction of host tryptophan metabolite kynurenic acid were significantly raised in model mice administrated with magnolol and infliximab (Figure 5). Furthermore, magnolol treatment (10 mg/kg) significantly elevated the microbial tryptophan metabolites including indolelactic acid, 5-hydroxyindoleacetic acid, 3-indolepropionic acid, indoxylsulfuric acid and indoleacetic acid in the colitis mice model, rather than infliximab (Figure 5).

## 3. Discussion

Magnolol is not only an anti-inflammatory agent but also a modulator on gastrointestinal motility, which is supported by the findings that: (1) magnolol suppresses pro-inflammatory cytokines and modulates the related pathways in vitro [23,24,25]; (2) magnolol exhibits significant therapeutic effects on animal models with different inflammatory diseases [11,13,14,26]; (3) magnolol exerts inhibitory effects on the smooth muscle contraction of gastric, ileum and colon [19,27]; (4) interestingly, magnolol shows stimulatory effects on gastrointestinal motility at lower dosages [22,28]; (5) magnolol regulates the calcium-activated potassium channels signaling pathway in the enterotoxigenic Escherichia coli-induced diarrhea mice [29]. These evidences indicate that magnolol have potential value in the treatment of gastroenterology disorders caused by intestinal inflammation or diarrhea such as colitis. Here in this study, we demonstrated for the first time experimental evidence suggesting that magnolol effectively improved the symptoms of DSS-induced colitis in mice. The therapeutic effect of magnolol is reflected in the obvious manifestations that diarrhea and rectal bleeding in DSS-induced mice were significantly attenuated by magnolol treatment, accompanied by recovered body weight loss, comparable with infliximab administration. Besides bloody diarrhea, DSS also induced mucosal ulceration with inflammatory cell infiltrate and crypt loss which are common characters in ulcerative colitis (UC) patients [30]. Magnolol showed fine improvement on DSS-induced inflammatory cell infiltrate and damage in mice colon, as well as substantial changes of colonic pro-inflammatory cytokine levels. The results suggest that magnolol might be a good drug candidate than for UC treatment.

The past few years have seen a rapid increase in studies of the assessment of metabolite profiles on experimental and human inflammatory bowel disease (IBD). Metabolomics is predicted to be an important manifestation in the diagnosis and management of IBD [31]. Therefore, we performed the untargeted metabolomics using UPLC/MS. Upon DSS-induction with or without drug treatment, the serum metabolites in mice showed distinct clustering and a clear separation of the control group, DSS-treated group, infliximab-treated group and magnolol-treated groups. It was noted that the metabolites of tryptophan metabolism, arginine and proline metabolism, bile acid biosynthesis, unsaturated fatty acid synthesis, arachidonic acid metabolism, and microbial biotransformation were changed by DSS-treatment, partly consistent with the previous findings in colitis mice model [32,33,34,35,36]. Intriguingly, magnolol and infliximab exhibited diverse effects in modulating the changed metabolites caused by colitis, due to different mechanisms of action. As a monoclonal antibody against TNF-α, infliximab was effective in regulating cholic acid, citric acid, hydroxyoctadecadienoic acid, p-cresol glucuronide, glucuronic acid, and kynurenic acid. In comparison, magnolol significantly resumed the changes of fatty acids, citric acid, d-Fructose, sphingolipid, polyamine, amino acids and tryptophan metabolites induced by DSS.

Tryptophan is an essential component of the human diet widely used in numerous research and clinical trials for its critical roles in metabolic functions regulating inflammation [37]. As a constituent of protein synthesis, tryptophan metabolism also involves two important metabolic pathways, serotonin (5-HT) synthesis and kynurenine synthesis [38]. Regulated by a rate-limiting enzyme, tryptophan hydroxylase (TPH), tryptophan is converted to 5-HT, playing important roles in the endocrine system and metabolism. In general, roughly 95% of total circulating serotonin is released by intestinal enterochromaffin cells in the gastrointestinal tract, maintaining the functions of pain control, pancreatic enzyme secretion, intestinal motility and visceral hypersensitivity, etc. [39,40]. On the other hand, kynurenine is produced from tryptophan by the action of tryptophan-2,3-dioxygenase (TDO) or the indoleamine-2,3-dioxygenase (IDO), which is the dominant physiological pathway for tryptophan [41]. It is known that kynurenine derived from host metabolism and indole-derived tryptophan metabolites produced by gut microbiota are endogenous ligands of aryl hydrocarbon receptor (AHR), an important regulator of immune response [42]. AHR disruption results in disordered immune responses, including lowered Treg cell levels, increased TNF-α levels, and a modified timeframe of IL-10 and IL-12 secretion [43]. In DSS-induced colitis mice, dysbiosis alters tryptophan metabolites production, and in turn, lessens the activation of AHR, affecting the host’s immune response and disrupting intestinal homeostasis [44,45]. Combined with the fact that AHR activators can suppress IL-6 expression by bone marrow stromal cells, and ameliorate DSS-induced acute colitis by increasing IL-10 and suppressing IFN-γ expression [46,47], correcting impaired microbiota functions to improve AHR ligands production is a promising strategy in IBD. Here we confirmed that the tryptophan metabolites including kynurenic acid, 5-hydroxyindoleacetic acid, indoleacetic acid, indolelactic acid, and indoxylsulfuric acid were significantly suppressed in DSS-induced colitis mice. Interestingly, infliximab was found to specifically regulate host-derived kynurenic acid production in the DSS model, whereas magnolol can act on both host-derived kynurenic acid and indole-derived tryptophan metabolites from microbial metabolism. These results indicate that magnolol can ameliorate DSS-induced acute colitis by rejuvenating tryptophan metabolism of host and microbiome to trigger colonic AHR activation.

**Figure 6:**
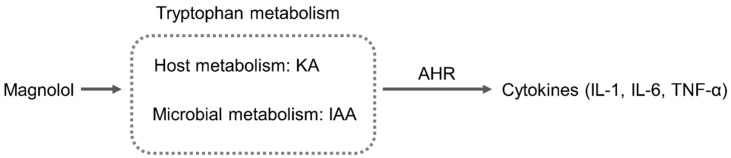
The schematic in the anti-inflammatory effect of magnolol on DSS-induced colitis.

In summary, tryptophan metabolism plays important roles in the pathogenesis and therapeutics of IBD. Results in this work demonstrated that magnolol exerted anti-inflammatory effect in DSS-induced colitis in mice. The underlying mechanisms may be associated with the enhancement of AHR activation by increasing tryptophan metabolites production that is suppressed by colonic inflammation (Figure 6).

## 4. Materials and Methods

### 4.1. Animals

Male C57BL/6 mice weighing about 20~25 g (8-week-old) were purchased from the Laboratory Animal Services Center, the Chinese University of Hong Kong, Hong Kong. The animals were fed with a rodent standard diet with free access water *ad libitum* and were housed in rooms maintained at 22 ± 1 °C with a 12 h light/dark cycle. The Animal Ethics Committee of School of Chinese Medicine, Hong Kong Baptist University, approved all experimental protocols, in accordance with “Institutional Guidelines and Animal Ordinance” (Department of Health, Hong Kong Special Administrative Region).

### 4.2. Chemicals and Reagents

Magnolol (purity > 96%) was purchased from NanJing TCM Institute of Chinese Materia Medica (NanJing, China). Dextran sulfate sodium (DSS; molecular weight: 36,000 to 50,000) was obtained from MP Biomedicals (Santa Ana, CA, USA). Mouse TNF-α, IL-1β, and IL-6 ELISA kits were purchased from eBioscience (San Diego, CA, USA). Hexadecyltrimethylammonium bromide (CTAB), hematoxylin, eosin, o-dianisidine dihydrochloride, and hydrogen peroxide (H_2_O_2_) were obtained from Sigma-Aldrich (St. Louis, MO, USA).

### 4.3. Colitis Model and Drug Treatment

Experimental colitis mice model was induced by routine administration of DSS solution dissolved in drinking distilled water at a concentration of 2.0% (*w*/*v*) *ad libitum* for 5 consecutive days as previously described by Wirtz [48]. Distilled water was given to mice in the normal group for the same period. The body weight of each mice was recorded daily in the morning (9:00 a.m.). On day 6, the mice with significant body weight loss, diarrhea, and gross bleeding were considered as experimental candidates of colitis. All the mice with comparable disease index were then randomly divided into 5 groups (*n* = 8/group): (1) DSS model group, intragastric administrated with saline; (2) positive control group, intraperitoneal injected with infliximab (5 mg/kg); (3) low dose treatment group, intragastric administrated with magnonol (5 mg/kg); (4) medium dose treatment group, intragastric administrated with magnonol (10 mg/kg); (5) high dose treatment group, intragastric administrated with magnonol (15 mg/kg). The mice in control group received drinking water without DSS throughout the entire experimental period and intragastric administrated with saline.

### 4.4. Disease Activity Index Evaluation

Body weight, stool consistency and fecal blood were monitored daily from the first day of drug treatment. Disease activity index (DAI) was determined as described previously [49,50]. Briefly, the scores of body weight loss, stool consistency and stool blood were calculated based on standard parameters [49]. Fecal occult blood was detected with the hemoccult sensa slides according to the manufacturer’s protocols (Beckman Coulter, Inc., Brea, CA, USA). Colon length of each mice were measured at the end of the experiment.

### 4.5. Hematoxylin/Eosin Staining

Mice were euthanized with CO_2_ and the colons were dissected, followed by a gentle washing with ice-cold phosphate buffered saline (PBS). Distal colons with 0.5~1 cm length were fixed in 4% paraformaldehyde for 12 h and embedded in paraffin. Colon sections were stained with hematoxylin/eosin and then subjected to blind analysis and scored as previously described [49,50].

### 4.6. Myeloperoxidase Activity Assay

To evaluate the severity of colon inflammation, myeloperoxidase (MPO), an enzyme mainly released by neutrophil, was examined in mice proximal colon as described previously [49]. Briefly, 100 mg colon tissue was isolated and homogenized in 1 mL 0.5% hexadecyltrimethylammonium bromide. Supernatant was separated by centrifugation and mixed with appropriate volume of potassium phosphate buffer (50 mmol, pH 6.0) with 0.0005% o-dianisidine dihydrochloride and 0.1% hydrogen peroxide. Changes in optical density were measured at 460 nm at room temperature (25 °C). MPO activity was calculated from the rate of optical density changes, and one unit of MPO activity was defined as the amount of enzyme present that produced a change in optical density of 1.0 U/min at 25 °C in the final reaction volume. The results were normalized to the total protein amount of colon tissues and quantified as units/mg protein.

### 4.7. Measurement of Colonic Levels of Cytokines

Mice colonic tissues (100 mg) were isolated and homogenized with 100 μL RIPA lysis buffer supplemented with protease inhibitor cocktail (Roche, Mannheim, Germany). The supernatant was collected as total protein samples after centrifugation at 13,000 rpm at 4 °C. Protein concentrations were determined using Pierce BCA Protein Assay Kit (Thermo Fisher Scientific Inc., Waltham, MA, USA). Levels of IL-1, IL-6 and TNF-α in the homogenates were examined using ELISA kits according with the standard procedures recommended by eBioscience company. The results were normalized to the total weight of colon tissues and quantified as pg/mg.

### 4.8. UPLC Separation of Serum Metabolites

Serum metabolites was extracted using the liquid–liquid method [51]. Four-fold volume of methanol was used to 50 μL of serum. After vortex and centrifugation, the resulting supernatants were mixed with 10 μL of L-4-chlorophenylalanine solution (0.2 mg/mL) as internal standard (IS) for metabolic profiling. An ultrahigh-performance liquid chromatography system (UHPLC, Agilent 1290 Infinity, Santa Clara, CA, USA) was used for separation of endogenous metabolome using a 1.7 μm C18 column (2.1 mm × 50 mm, Waters, Milford, MA, USA). The mobile phases consist of water with 0.1% formic acid (A) and acetonitrile with 0.1% formic acid (B). The gradient program was as follows: starting from 5% B and progressing to 35% B in 4 min, 50% B in 2 min, and then 100% B in 6 min, holding at 100% B for 3 min, finally returning back to 5% B and equilibrating in 2 min. The separated components were subsequently fragmented and analyzed using a mass spectrometer.

### 4.9. QTOF-MS Analysis and Metabolite Identification

A quadrupole time-of-flight mass spectrometer (QTOF-MS, Agilent 6543, Santa Clara, CA, USA) was coupled with electrospray ionization source for fragmental collection. For full scan MS analysis, the QTOF-MS conditions were set as follows: the temperature for desolvation gas, 300 °C; gas flow, 8 L/min. The capillary voltage and cone voltage were set to 3.2 kV and 35 V for ESI+, and 3 kV and 50 V for ESI−, respectively. The mass range was set from 80 to 1000 *m*/*z*. The scan time was set at 0.3 s, and the inter scan delay was set at 0.02 s. Under the target MS/MS acquisition mode, the MS/MS range was set from 30 to 800 *m*/*z* and the collision energies were set as 10 eV, 20 eV and 40 eV for comparing with chemical standards obtained from Sigma-Aldrich (St. Louis, MO, USA) or METLIN database. Under the target MS/MS mode, other parameters mentioned above are the same as settings in the full scan mode.

### 4.10. Statistical Analysis

For metabolomic analysis, the raw MS data was filtered and aligned using R (Version 3.1.2, University of Auckland, Auckland, New Zealand) with XCMS package [52]. SIMCA-P software (Version 11.0, Sartorius Stedim Biotech, Goettingen, Germany) were utilized for multivariate statistical analysis (MSA) and plotting scatter diagrams of MSA. Metabolic features that characterized as variable importance in the projection (VIP) > 1 and adjusted *p* value < 0.1 [53], were selected for pathway enrichment analysis referred to the databases of KEGG and SMPDB. In other statistical analysis, the data was evaluated as means ± standard error of the mean (SEM). Statistical differences between individual groups were evaluated using Student’s *t*-test or one-way analysis of variance (ANOVA). All experiments were performed at least three times independently. GraphPad Prism 6.0 software (GraphPad Software Inc., San Diego, CA, USA) was used for the calculations, and *p* < 0.05 was considered statistically significant.

## Figures and Tables

**Table 1:** Biomarkers identified in the serum of mice induced by DSS.

N.	ESI	R.T.	*m*/*z*	Metabolite	Pathway Classification
1	-	8.97	319.2285	8-HETE	Arachidonic acid metabolism
2	-	10.89	303.2334	Arachidonic Acid	Arachidonic acid metabolism
3	-	6.40	407.2810	Cholic acid	Bile acid biosynthesis
4	-	7.72	391.2861	Deoxycholic acid	Bile acid biosynthesis
5	-	8.40	317.2126	18-HEPE	Biosynthesis of unsaturated fatty acid
6	-	8.87	343.2285	17-HDoHE	Biosynthesis of unsaturated fatty acid
7	-	10.30	301.2178	Eicosapentaenoic Acid	Biosynthesis of unsaturated fatty acid
8	-	0.82	191.0199	Citric acid	Citrate cycle
9	-	0.56	179.0558	d-Fructose	Fructose and mannose metabolism
10	-	8.57	295.2282	9-HODE	Linoleic acid metabolism
11	-	1.70	241.0831	Equol	Microbial isoflavones biotransformation
12	-	2.46	181.0509	Hydroxyphenyllactic acid	Microbial tyrosine biotransformation
13	-	3.55	283.0826	p-Cresol glucuronide	Microbial tyrosine biotransformation
14	-	0.59	195.0506	Gluconic acid	Pentose phosphate pathway
15	-	5.02	321.0445	dTMP	Pyrimidine metabolism
16	-	7.77	378.2422	Sphingosine-1-phosphate	Sphingolipid metabolism
17	-	2.62	190.0543	5-Hydroxyindoleacetic acid	Tryptophan metabolism
18	-	3.71	212.0027	Indoxylsulfuric acid	Tryptophan metabolism
19	-	4.05	204.0669	Indolelactic acid	Tryptophan metabolism
20	+	0.61	132.0775	Creatine	Arginine and proline metabolism
21	+	0.56	175.1212	l-Arginine	Arginine and proline metabolism
22	+	10.46	245.2275	N1-Acetylspermine	Arginine and proline metabolism
23	+	8.11	516.3067	Taurocholic acid	Bile acid biosynthesis
24	+	0.60	161.1364	Propionylcholine	Glycerophospholipid metabolism
25	+	0.50	147.1104	l-Lysine	Lysine degradation
26	+	0.79	123.0548	Niacinamide	Nicotinate and nicotinamide metabolism
27	+	0.59	137.0726	*N*-methylnicotinamide	Nicotinate and nicotinamide metabolism
28	+	1.69	127.0508	Thymine	Pyrimidine metabolism
29	+	5.18	345.2066	11-Dehydrocorticosterone	Steroid hormone biosynthesis
30	+	4.77	190.0861	3-Indolepropionic acid	Tryptophan metabolism
31	+	0.57	176.0668	Indoleacetic acid	Tryptophan metabolism
32	+	2.81	190.0501	Kynurenic acid	Tryptophan metabolism
33	+	0.97	182.0759	l-Tyrosine	Tyrosine metabolism
34	+	0.60	118.0875	l-Valine	Valine, leucine and isoleucine metabolism

**Table 2:** Significant changes of metabolome in colon tissue of mice induced by DSS.

Metabolite Name	CON	DSS	5 mg/kg	10 mg/kg	15 mg/kg	POS
8-HETE	0.433 ± 0.092	2.283 ± 0.623	1.234 ± 0.415	0.568 ± 0.096 *	1.218 ± 0.332	1.496 ± 0.621
Arachidonic Acid	1.223 ± 0.196	0.708 ± 0.079	0.972 ± 0.184	0.809 ± 0.130	0.814 ± 0.117	0.633 ± 0.116
Cholic acid	0.498 ± 0.094	0.898 ± 0.107	0.673 ± 0.125	2.087 ± 0.507 *	2.192 ± 0.547 *	0.424 ± 0.164 *
Deoxycholic acid	0.325 ± 0.039	0.200 ± 0.027	0.145 ± 0.039	0.447 ± 0.190	0.272 ± 0.079	0.197 ± 0.070
18-HEPE	0.435 ± 0.115	1.883 ± 0.498	0.759 ± 0.237	0.381 ± 0.085 *	0.704 ± 0.197 *	1.249 ± 0.581
17-HDoHE	0.559 ± 0.142	2.045 ± 0.553	0.782 ± 0.353	0.195 ± 0.072 *	0.613 ± 0.167 *	0.898 ± 0.449
Eicosapentaenoic Acid	1.421 ± 0.272	0.594 ± 0.081	0.817 ± 0.164	0.729 ± 0.148	0.623 ± 0.115	0.551 ± 0.109
Citric acid	5.263 ± 0.925	2.982 ± 0.545	6.155 ± 1.213 *	6.280 ± 0.640 *	5.226 ± 0.726 *	5.886 ± 0.700 *
d-Fructose	0.326 ± 0.019	0.203 ± 0.017	0.290 ± 0.034 *	0.252 ± 0.022	0.251 ± 0.048	0.240 ± 0.025
9-HODE	0.255 ± 0.054	1.001 ± 0.281	0.354 ± 0.108 *	0.202 ± 0.041 *	0.233 ± 0.058 *	0.309 ± 0.107 *
Equol	0.245 ± 0.018	0.186 ± 0.024	0.139 ± 0.020	0.191 ± 0.023	0.140 ± 0.017	0.171 ± 0.022
Hydroxyphenyllactic acid	0.484 ± 0.078	0.195 ± 0.048	0.496 ± 0.134	0.386 ± 0.072 *	0.444 ± 0.078 *	0.479 ± 0.094 *
p-Cresol glucuronide	0.221 ± 0.059	0.810 ± 0.300	0.285 ± 0.069	0.280 ± 0.087	0.311 ± 0.094	0.124 ± 0.024 *
Gluconic acid	0.253 ± 0.034	0.072 ± 0.009	0.161 ± 0.039 *	0.121 ± 0.014 *	0.161 ± 0.031 *	0.127 ± 0.019 *
dTMP	3.155 ± 0.363	1.516 ± 0.384	1.543 ± 0.413	4.627 ± 0.579 *	2.014 ± 0.500	1.894 ± 0.486
Sphingosine-1-phosphate	0.645 ± 0.046	0.513 ± 0.032	0.676 ± 0.083	0.744 ± 0.064 *	0.710 ± 0.060 *	0.512 ± 0.040
5-Hydroxyindoleacetic acid	0.146 ± 0.030	0.064 ± 0.015	0.098 ± 0.014	0.170 ± 0.025 *	0.094 ± 0.022	0.090 ± 0.020
Indoxylsulfuric acid	7.599 ± 1.098	4.970 ± 0.765	4.024 ± 0.806	8.662 ± 1.511 *	4.378 ± 0.787	4.752 ± 0.856
Indolelactic acid	0.515 ± 0.091	0.164 ± 0.044	0.248 ± 0.082	0.526 ± 0.038 *	0.276 ± 0.059	0.191 ± 0.048
Creatine	1.277 ± 0.239	0.710 ± 0.147	1.061 ± 0.187	1.365 ± 0.217 *	1.159 ± 0.357	0.960 ± 0.193
l-Arginine	0.242 ± 0.035	0.162 ± 0.009	0.154 ± 0.014	0.200 ± 0.023	0.153 ± 0.030	0.160 ± 0.018
N1-Acetylspermine	0.046 ± 0.014	0.016 ± 0.004	0.056 ± 0.019	0.076 ± 0.016 *	0.045 ± 0.009 *	0.022 ± 0.008
Taurocholic acid	0.538 ± 0.043	0.742 ± 0.108	0.453 ± 0.073 *	0.640 ± 0.088	0.532 ± 0.104	0.673 ± 0.151
Propionylcholine	0.148 ± 0.013	0.091 ± 0.006	0.091 ± 0.009	0.090 ± 0.008	0.098 ± 0.009	0.100 ± 0.008
l-Lysine	0.086 ± 0.007	0.061 ± 0.005	0.097 ± 0.011 *	0.088 ± 0.006 *	0.089 ± 0.011 *	0.079 ± 0.005
Niacinamide	0.642 ± 0.074	0.472 ± 0.063	0.581 ± 0.055	0.444 ± 0.035	0.533 ± 0.052	0.482 ± 0.043
*N*-methylnicotinamide	0.068 ± 0.010	0.138 ± 0.031	0.221 ± 0.047	0.116 ± 0.016	0.187 ± 0.028	0.152 ± 0.019
Thymine	0.069 ± 0.005	0.044 ± 0.006	0.034 ± 0.005	0.047 ± 0.008	0.034 ± 0.005	0.037 ± 0.006
11-Dehydrocorticosterone	0.012 ± 0.001	0.035 ± 0.005	0.058 ± 0.012	0.034 ± 0.004	0.079 ± 0.016 *	0.047 ± 0.010
3-Indolepropionic acid	0.064 ± 0.007	0.032 ± 0.003	0.033 ± 0.006	0.036 ± 0.008	0.021 ± 0.002 *	0.047 ± 0.007
Indoleacetic acid	0.041 ± 0.004	0.028 ± 0.003	0.037 ± 0.003	0.040 ± 0.004 *	0.041 ± 0.005 *	0.036 ± 0.005
Kynurenic acid	0.073 ± 0.016	0.022 ± 0.005	0.058 ± 0.013 *	0.065 ± 0.009 *	0.050 ± 0.009 *	0.065 ± 0.009 *
l-Tyrosine	3.308 ± 0.338	1.742 ± 1.344	2.024 ± 0.302	3.071 ± 0.326 *	2.057 ± 0.238	2.665 ± 0.495
l-Valine	1.305 ± 0.107	0.951 ± 0.082	1.072 ± 0.176	1.329 ± 0.096 *	1.184 ± 0.221	1.157 ± 0.196

Intensity of metabolite is expressed as mean ± SEM (*n* = 8/group); Superscript: *, Fold-change and *T*-test value is calculated after comparison between magnolol-treated (or infliximab-treated) group and DSS group.
